# Sport and Exercise Therapy in the Treatment of Mental Illness -Let’s get moving !

**DOI:** 10.1192/j.eurpsy.2025.1821

**Published:** 2025-08-26

**Authors:** K. Friedrich, C. A. Penkov, J. Krieger, V. Rößner-Ruff, M. Wendt, M. Ziegenbein

**Affiliations:** 1 Forschung & Entwicklung; 2 Sporttherapie, Wahrendorff Klinikum, Sehnde, Germany

## Abstract

**Introduction:**

As a complementary or stand-alone treatment, sport and exercise therapy (SET) can have a therapeutic effect on the symptoms of mental illness as well as having a therapeutic or preventive effect on physical comorbidities. Therefore, treatment guidelines recommend the integration of exercise therapy as a complementary approach in a multimodal treatment. In a clinical inpatient setting SET are supervised by specialised professionals and conducted in an individual or group setting. Low level of participation in SET during treatment points to the need for research into influencing factors. One study suggests, that SET during mental health treatment increases the likelihood of meeting the physical activity recommendations.

**Objectives:**

The purpose of the study is to investigate the extent to which SET can increase patients’ levels of physical activity during inpatient treatment and, in particular, promote a physically active lifestyle after inpatient treatment, thereby supporting long-term stabilisation. In order to gain further insight into the factors that influence participation in SET during treatment, this study analyses both intrapersonal and interindividual factors.

**Methods:**

Patients (age ≥ 18 years, all genders) in partial- or full-time inpatient treatment at a psychotherapeutic and psychosomatic specialist clinic in Lower Saxony, Germany are examined by online self-report questionnaire. It’s a longitudinal study design with 3 time points (start of treatment, end of treatment, 12 weeks after the end of treatment). Patients participate in SET as part of their treatment. Physical activity in minutes per week and the therapeutic alliance between exercise therapists and patients are measured. In addition, self-efficacy expectations, sport- and exercise-related self-concordance and the subjectively perceived effectiveness of SET are assessed as further factors influencing physical activity.

**Results:**

The results of the inferential-static data analysis will be reported.

**Conclusions:**

Based on the results, possible implications for the focus of SET and the role of exercise therapists are discussed. Conclusions based on motivational aspects of maintaining a physically active lifestyle after the end of treatment are considered.

**Disclosure of Interest:**

None Declared

